# The Osteogenic Properties of Multipotent Mesenchymal Stromal Cells in Cultures on TiO_2_ Sol-Gel-Derived Biomaterial

**DOI:** 10.1155/2015/651097

**Published:** 2015-02-01

**Authors:** Krzysztof Marycz, Agnieszka Śmieszek, Jakub Grzesiak, Anna Siudzińska, Monika Marędziak, Anna Donesz-Sikorska, Justyna Krzak

**Affiliations:** ^1^Electron Microscopy Laboratory, University of Environmental and Life Sciences, Kożuchowska 5b, 50-631 Wroclaw, Poland; ^2^Wrocław Research Centre EIT+, Stablowicka 147, 54-066 Wroclaw, Poland; ^3^Institute of Materials Science and Applied Mechanics, Wroclaw University of Technology, Smoluchowskiego 25, 50-370 Wroclaw, Poland

## Abstract

The biocompatibility of the bone implants is a crucial factor determining the successful tissue regeneration. The aim of this work was to compare cellular behavior and osteogenic properties of rat adipose-derived multipotent stromal cells (ASCs) and bone marrow multipotent stromal cells (BMSCs) cultured on metallic substrate covered with TiO_2_ sol-gel-derived nanolayer. The morphology, proliferation rate, and osteogenic differentiation potential of both ASCs and BMSCs propagated on the biomaterials were examined. The potential for osteogenic differentiation of ASCs and BMSCs was determined based on the presence of specific markers of osteogenesis, that is, alkaline phosphatase (ALP), osteopontin (OPN), and osteocalcin (OCL). Additionally, the concentration of calcium and phosphorus in extracellular matrix was determined using energy-dispersive X-ray spectroscopy (SEM-EDX). Obtained results showed that TiO_2_ layer influenced proliferation activity of ASCs, which manifested by shortening of population doubling time and increase of OPN secretion. However, characteristic features of cells morphology and growth pattern of cultures prompted us to conclude that ultrathin TiO_2_ layer might also enhance osteodifferentiation of BMSCs. Therefore in our opinion, both populations of MSCs should be used for biological evaluation of biomaterials compatibility, such results may enhance the area of investigations related to regenerative medicine.

## 1. Introduction

The biocompatibility of bone implants is a crucial factor determining the successful tissue regeneration. In the field of orthopaedic and oral medicine, metallic materials have found wide application both as permanent and temporary devices. Among various metallic prostheses, the stainless steel 316 L grade is most commonly used material in modern orthopaedics and dentistry. It is often applied as plates or nails, used preferably due to the proper mechanical properties and because of its low cost [1]. The main disadvantage of stainless steel 316 L is its corrosion resistance that decreases in contact with body fluids. Metals ions released from stainless steel have systemic effect and may negatively affect immunity and nervous and endocrine system [2]. Additionally, compounds of stainless steel 316 L such as chromium and nickel are also considered as potentially carcinogenic factors [3]. Currently, the surface of commonly used implants is improved by applying various types of oxide coatings, maintaining fine biophysical properties of austenitic steel 316 L and protecting it from oxidative action of body fluids [4–6]. One of the most promising methods for improvement of metallic biomaterials surfaces is a sol-gel route. It has been already used for the synthesis of various advanced biomedical implants for clinical application [7–9]. The sol-gel-derived layers not only enhance corrosion resistance of stainless steel but also improve its biocompatibility [10, 11]. In our previous research, we developed and analyzed the 316 L austenitic steel implant covered with TiO_2_ layer using sol-gel method. These results clearly demonstrated biocompatibility of obtained biomaterials, and its functionality manifested with proper integration of implants with the tissue [12]. As it was shown titanium dioxide increases the adhesion of bone precursor cells and therefore may accelerate the formation of new bone [13]. However, novel perspective for the evaluation of biocompatibility of bioimproved materials involves application of mesenchymal stem/stromal cell populations (MSCs), especially as they are used as a therapeutic strategy in order to improve the processes of tissue regeneration [14]. Stromal cells derived from adipose tissue (ASCs) and bone marrow (BMSCs) are to date most accurately described. They are widely considered as a significant indicator of the biocompatibility and osteogenic properties of the material due to their multipotent abilities to differentiate into bone precursor cells [5, 8, 9]. These two populations differ in their origin and in some specific features, like the susceptibility to osteogenic differentiation or self-renewal properties; however, both are proposed for therapeutic usage concurrently, mainly because high similarity of morphology and surface antigens [15, 16]. The screening of particular biomaterial for medical purposes should include an analysis of the biological response of both ASCs and BMSCs. The morphology of cells, growth pattern of culture, and the proliferation rate of mesenchymal stromal cells in cultures with biomaterials might serve as very useful parameters for evaluation of biocompatibility of biomaterials, particularly in the context of further* in vivo* studies.

The integration of biomaterial with the surrounding tissue is crucial for proper healing and complete recovery of the patient. Therefore, when considering metallic biomaterials for orthopedic purposes, the osteogenic potential of MSCs should also be taken into account, as we stated previously [8, 9]. Assessment of response and cytophysiology of cells in the culture on investigated surfaces may provide essential information regarding not only its toxicity but also potential biofunctionality of biomaterial. However, in this regard the biology of ASCs and BMSCs is still poorly investigated and needs further detailed investigations.

The purpose of this work was to determine the osteogenic properties of both ASCs and BMSCs cultured on 316 L stainless steel-based biomaterial covered with TiO_2_ sol-gel layer. In our model, special attention has been paid to determining proliferating activity of BMSCs and ASCs. The degree of MSCs differentiation towards bone-like tissue was evaluated based on the activity of specific osteogenic factors: alkaline phosphatase (ALP), osteopontin (OPN), and osteocalcin (OCL) [17]. Alkaline phosphatase assay combined with the determination of calcium and phosphorus deposition was used as a tool for the identification of matrix mineralization, while noncollagenous proteins of bone matrix, that is, OPN and OCL, were detected at the intracellular and extracellular levels.

## 2. Materials and Methods

### 2.1. Synthesis of TiO_2_ Coatings

For the synthesis of sol-gel-derived TiO_2_ coatings, the following substrates were used: titanium(IV) ethoxide (TiEO; Alfa Aesar, Karlsruhe, Germany) as titanium precursor, ethanol (99% EtOH, POCh S.A., Gliwice, Poland) as a solvent, and acetylacetone as a complexing agent of titanium precursor (AcAc, Sigma Aldrich, Munich, Germany) in molar ratio 1 : 11.4 : 2.1. The hydrolysate was deposited on stainless austenitic steel 316 L discs (15 mm in diameter, consistent with the diameter of culture well) by dip-coating method, with a controlled process parameters, that is, dipping and pulling speed (*v* = 34.26 mm/min) and the residence time of the substrate in the hydrolysate (1st layer: 60 s, 2nd layer: 30 s, and 3rd layer: 15 s). This process was repeated three times to obtain three-layer coatings. Afterwards, coating was dried at room temperature (22°C ± 1°C) on air and then annealed for 12 hours at 250°C with controlled temperature gradient (1.5°C/min). Noncoated steel discs were also exposed to the same high temperature conditions (250°C for 12 hours).

### 2.2. Analysis of Surface Morphology and Chemical Composition

The morphology of 316 L stainless steel substrate as well as that of titanium dioxide layer, applied on biomaterials, was determined using scanning electron microscope (EVO LS15; Carl Zeiss, Jena, Germany) with a secondary electron detector (SE). Moreover, the energy-dispersive X-ray spectroscopy (EDX) was used to determine the chemical composition of coatings obtained and to evaluate the distribution of titanium dioxide. The analysis was carried out with the use of a Quantax detector (Brüker) and the images were captured at 500-fold magnification.

### 2.3. Isolation of Mesenchymal Stromal Cells from Adipose Tissue and Bone Marrow

Both ASCs and BMSCs were isolated from tissues derived from adult Wistar rats. Utility of animal tissues was approved by Second Local Ethic Commission (Wroclaw, Poland; Decision number 84/2012).

Adipose-derived mesenchymal stromal cells were isolated from rat subcutaneous fat tissue, collected from abdominal region. Adipose tissue fragments were mechanically minced and digested in type I collagenase for 40 minutes at 37°C, according to the procedure described previously [18, 19]. The homogenates were centrifuged for 10 minutes at 1200 ×g in order to obtain stromal-vascular fraction containing MSCs.

The BMSC population was isolated from the femoral bone marrow of rats. The marrow canal was rinsed with Hank's solution, using 20 G needle and syringe, following the procedure described previously [20]. The suspension of cells was centrifuged at 300 ×g for 4 minutes. Cells precipitates were suspended in primary culture medium and transferred to a suitable flask.

### 2.4. Culture and Expansion of Mesenchymal Stromal Cells

The isolated cells were resuspended in the culture medium and transferred to T25 culture flasks. The primary cultures were propagated in DMEM/Ham's F12 (Sigma Aldrich, Munich, Germany), whereas secondary cultures were maintained in DMEM with 4500 mg/L of glucose (Sigma Aldrich, Munich, Germany). Both media were supplemented with 10% of fetal bovine serum (FBS, Sigma Aldrich, Munich, Germany) and 1% of antibiotic/antimycotic solution. Cultures were maintained in CO_2_ incubator (5% CO_2_ and 95% humidity) with the temperature of 37°C. When 80–90% confluence was reached, cells were passaged using TrypLE Select solution (Life Technologies, Warsaw, Poland), according to the manufacturer's protocol. In order to obtain a sufficient number of cells for the test, cultures of both ASCs and BMSCs were passaged three times.

### 2.5. Phenotypic Characterization of Isolated Cells

Investigation of cells' phenotype included detection of the following markers: CD29, CD44, CD 45, CD73, and CD105. Antibodies used for the analysis were purchased from Sigma (mouse anti-rat integrin-b-1 (CD29), dilution 1 : 100; rabbit anti-rat NT5E (CD73), dilution 1 : 200; rabbit anti-rat endoglin (CD105), dilution 1 : 200; mouse anti-rat CD45, dilution 1 : 100), except for anti-CD44 obtained from R&D Systems (sheep anti-rat CD44, dilution 1 : 100). Secondary, fluorophore-labeled antibodies were purchased from Sigma (goat anti-mouse IgG atto-594, dilution 1 : 400; goat anti-rabbit IgG atto-488, dilution 1 : 400) and from R&D Systems (donkey anti-sheep IgG, NL557, dilution 1 : 400). Procedure of immunostaining was performed using general protocol described by the manufacturer. Incubation with primary antibodies was performed overnight, while reaction with secondary antibodies was performed at 37°C for 1 hour in dark. To establish background fluorescence and exclude the nonspecific staining, negative controls were performed in order to examine specificity of secondary antibody binding. Negative control samples were incubated with secondary antibody for 1 hour, at 37°C in dark. Additional specimens were also counterstained with 4′,6-diamidino-2-phenylindole (DAPI) to visualize nuclei. Samples were visualized using inverted fluorescence microscope (Axio Observer A1; Carl Zeiss, Jena, Germany). Images were captured with Canon PowerShot camera and merged using AxioVision 4.8 software (Carl Zeiss, Jena, Germany).

### 2.6. Multipotency Assay

Cultures of isolated cells were propagated in proper media designated for the differentiation of MSCs. StemPro adipogenesis and osteogenesis differentiation kits were purchased from Life Technologies and prepared according to the manufacturer's instructions. The stimulation of ASCs and BMSCs toward adipocytes lasted 14 days, while osteogenesis was induced during 21-day period. To evaluate adipogenic and osteogenic differentiation two specific staining methods were used, that is, Oil-Red O (Sigma Aldrich, Munich, Germany) for detection neutral lipid deposits and Alizarin Red (Sigma Aldrich, Munich, Germany) for calcium deposits. Preparations were analyzed using Axio Observer A1 inverted microscope (Axio Observer A1; Carl Zeiss, Jena, Germany), while the documentation was made using Cannon PowerShot camera.

### 2.7. *In Vitro* Test

For evaluation of osteogenic induction of ASCs and BMSCs in cultures with investigated biomaterials cells were propagated in 24-well plates, precoated with investigated biomaterials. Specimens were prepared in triplicate. The initial cell inoculum was equal to 8.5 × 10^4^ of MSCs per well in the presence of the growth medium (DMEM + 10% FBS). Stimulation with osteogenic medium began after four days (StemPro Osteogenesis Differentiation Kit, Life Technologies, Warsaw, Poland). The differentiation of both MSCs' populations was carried out for 18 days. Nonstimulated cultures were maintained in complete DMEM culture medium. Media were changed every 4 days.

### 2.8. Analysis of Cells Viability and Proliferation Ratio

The viability of cells was monitored with the resazurin-based cytotoxic assay. Measurement was performed every four days during media replacement. The collection of media was followed by the addition of a complete medium containing 10% of resazurin solution. The cultures were incubated with the dye for 2 hours. The supernatants were collected and transferred to 96-well microplates for spectrophotometric measurements. The absorbance was determined at 600 nm with 690 nm reference wavelength using spectrometer (BMG Labtech, Ortenberg, Germany). In order to determine the proliferation activity, population doubling time (PDT) was calculated. The algorithm for PDT was described previously [21] and in the current study was supported by the online software [22]. The number of cells used for the calculations was estimated based on the growth curve determined separately for ASCs and BMSCs.

### 2.9. Evaluation of Osteogenic Differentiation

#### 2.9.1. Alkaline Phosphatase Assay: ALP Assay

Extracellular activity of ALP was determined in the supernatants collected after 7, 14, and 18 days of osteogenic culture on investigated surfaces. The assay was performed with an Alkaline Phosphatase Colorimetric Assay Kit (Abcam, Cambridge, UK), according to the protocol attached by the manufacturer. The test samples were prepared from each culture in duplicate and were diluted twofold. Sample background control was included and the background was corrected by subtracting the value derived from the zero standards from all standards, samples, and sample background control. In the reaction, the p-nitrophenyl phosphate (pNPP) was used as a phosphatase substrate. The substrate was hydrolyzed into para-nitrophenol (pNP) by alkaline phosphatase. The product of the reaction was measured at 405 nm wavelength using spectrometer (BMG Labtech, Ortenberg, Germany). Sample readings were applied to the standard curve to obtain the amount of pNP generated by the ALP sample (glycine units). The enzymatic activity was determined using the following formula.

ALP activity (*μ*U/mL) = *A*/*V*/*T*, where (i) *A* is the amount of pNP generated by the samples (in *μ*mol); (ii) *V* is the volume of sample added to the assay well (in mL); and (iii) *T* is the reaction time.

#### 2.9.2. Quantification of Matrix Mineralization

The analysis of calcium and phosphorus deposited on investigated surfaces during the osteogenic differentiation was carried out using SEM-EDX technique. For this purpose, all investigated samples were fixated in 2.5% glutaraldehyde for 10 minutes at room temperature and dehydrated with graded series of ethanol. A Quantax detector (Brüker) was used for the analysis, with the parameters of 10 kV of filament tension and 11 mm of WD, using 200-fold magnification. From each sample three measurements were performed. The values obtained were presented as a weight percentage (wt%).

#### 2.9.3. Intracellular Detection of Osteogenic Stromal-Cell Maturation Markers: Immunofluorescence Staining

Two markers, that is, osteopontin (OPN) and osteocalcin (OCL), were visualized with specific antibodies, that is, rabbit anti-osteopontin IgG and mouse anti-osteocalcin purchased from Life Technologies (Poland). Immunofluorescent staining was performed according to the general procedures provided by the manufacturer. Osteogenic cultures were incubated with primary antibodies for 1 hour at 37°C (mouse anti-rat osteocalcin, dilution 1 : 100; rabbit anti-rat osteopontin, dilution 1 : 100; Sigma). Secondary antibodies were labeled with atto-488 (goat anti-rabbit IgG, dilution 1 : 400; Sigma) and with atto-594 (goat anti-mouse IgG, dilution 1 : 400; Sigma). Samples were incubated with secondary antibodies at 37°C for 1 hour in dark. Appropriate negative controls were performed by incubating the specimens with only secondary antibodies, for excluding the nonspecific reaction. Samples were also counterstained with 4′,6-diamidino-2-phenylindole (DAPI) to visualize nuclei. Specimens were analyzed with inverted fluorescence microscope (Axio Observer A1, Carl Zeiss, Jena, Germany). Images were captured with Cannon PowerShot camera and merged using AxioVision 4.8 software (Carl Zeiss, Jena, Germany).

#### 2.9.4. Detection of Secreted (Extracellular) Form of OPN and OCL with Enzyme-Linked Immunosorbent Assay (ELISA)

The investigated markers were detected in the supernatants collected after 18 days of each cell culture. The presence of OPN and OCL in the cell supernatants was determined using Mouse/Rat Osteopontin (OPN) Quantikine ELISA Kit (R&D Systems, Abingdon, UK) and Rat Gla-Osteocalcin High Sensitive EIA Kit (Takara, Otsu, Japan), respectively. Each sample was tested for the presence of specific markers in duplicate. The quantitative determination of OPN and OCL was performed according to the manufacturer's instructions. The amount of detected proteins was expressed as ng/mL of supernatant.

### 2.10. Statistical Analysis

The normality of the population data was determined using Shapiro-Wilk test, while equality of variances was assessed by Levene's test. Differences between groups were analyzed using parametric test (Student's *t*-test or analysis of variance (ANOVA)). Statistical analysis was performed with STATISTICA 10.0 software (StatSoft, Inc., Statistica for Windows, Tulsa, OK, USA). Differences with a probability of *P* < 0.05 were considered significant.

## 3. Results

### 3.1. Surface Characterization

**Figure 1:**
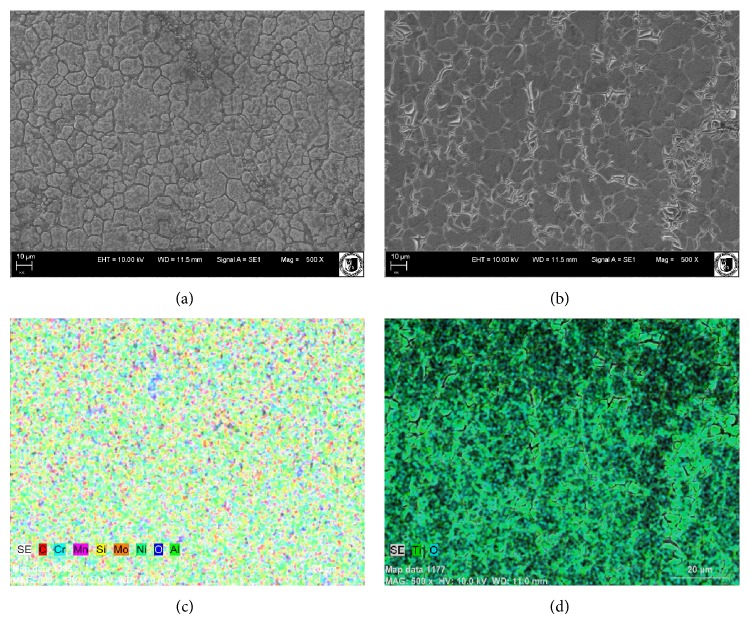
The results of SEM-EDX analysis. The surface morphology of 316 L stainless steel substrate (a) and 316 L stainless steel covered with TiO_2_ sol-gel-derived coating (b), combined with the analysis of elemental distribution. The SEM-EDX map of stainless steel (c) shows the distribution of its main chemical elements, and the map of the TiO_2_ coating (d) confirms the presence of Ti and demonstrates its deposition on the substrate's surface. Magnifications: 500x; SEM microphotographs were obtained using SE detector.

The surface of 316 L stainless steel was homogenous with visible characteristic grain boundaries (Figure 1(a)). Titanium dioxide coatings applied on 316 L stainless steel were transparent and homogenous, with robust structure characteristic for TiO_2_ (Figure 1(b)). The SEM-EDX analysis revealed the presence and uniform distribution of titanium in the synthesized coating (Figure 1(d)). The analysis of elemental composition performed for 316 L stainless steel showed characteristic image of elements' distribution, typical for this metallic substrate. As expected, in 316 L stainless steel, predominant elements were chrome (Cr), nickel (Ni), and molybdenum (Mo) (Figure 1(c)).

### 3.2. Phenotypic Characterization of Cells Used for the Test and Their Multipotent Properties

**Figure 2:**
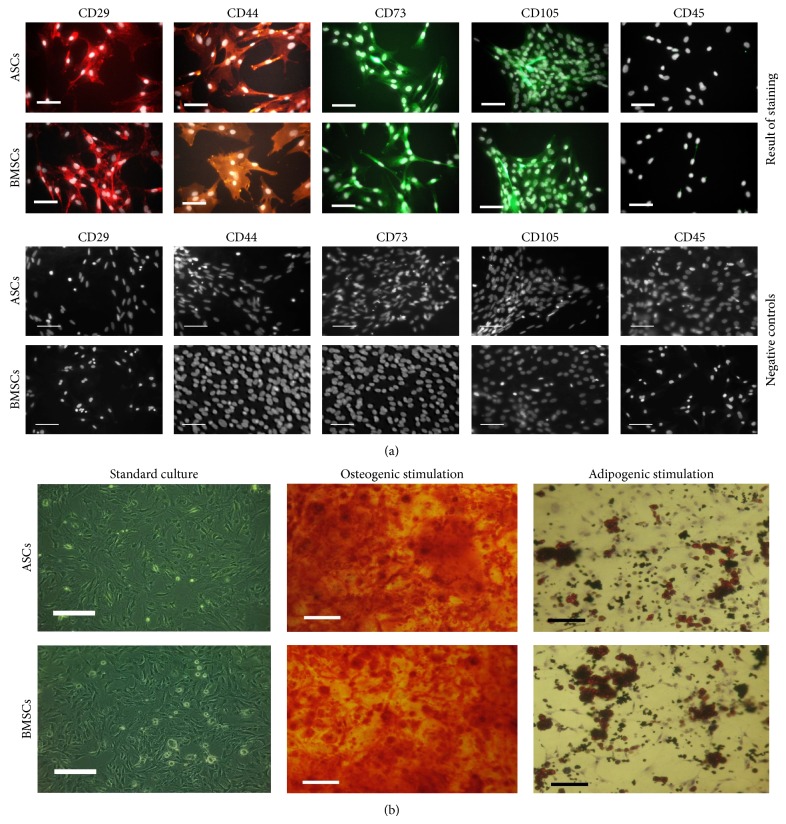
The results of phenotypic characterization of cells used for the test (a) and the results of their osteogenic and adipogenic differentiation (b). (a) Images show positive reaction with specific antibodies detecting CD29, CD44, CD73, and CD105, respectively. No specific reaction was noted with anti-CD45 antibodies. DAPI staining was used to show localization of nuclei, presented in the figures as white dots. Negative controls are presented to show specificity of performed staining. Scale bar = 50 *μ*m. (b) Images present morphology of rat ASCs and BMSCs cultured in DMEM medium supplemented with 10% of FBS (standard culture) and results of its osteogenic and adipogenic stimulation. For visualization of calcium deposits specific Alizarin Red staining was used, while lipid drops were stained using Oil-Red O. Scale bar = 200 *μ*m.

Performed analysis showed that both populations of cells, which were isolated according to the methods described, expressed markers of characteristic for multipotent mesenchymal stromal cells, that is, CD29, CD44, CD73, and CD105. Cells did not express marker specific for hematopoietic cell lineage, that is, CD 45 (Figure 2(a)). Additionally, the multipotent character of cells derived from adipose tissue and bone marrow of adult Wistar rats was confirmed by their ability for differentiation into bone cells and adipocytes. Specific staining showed that stimulation of ASCs and BMSCs with adipogenic medium promotes formation of lipid droplet, while induction of osteogenesis resulted in calcium-rich deposits development (Figure 2(b)).

### 3.3. Proliferation of Cells

**Figure 3:**
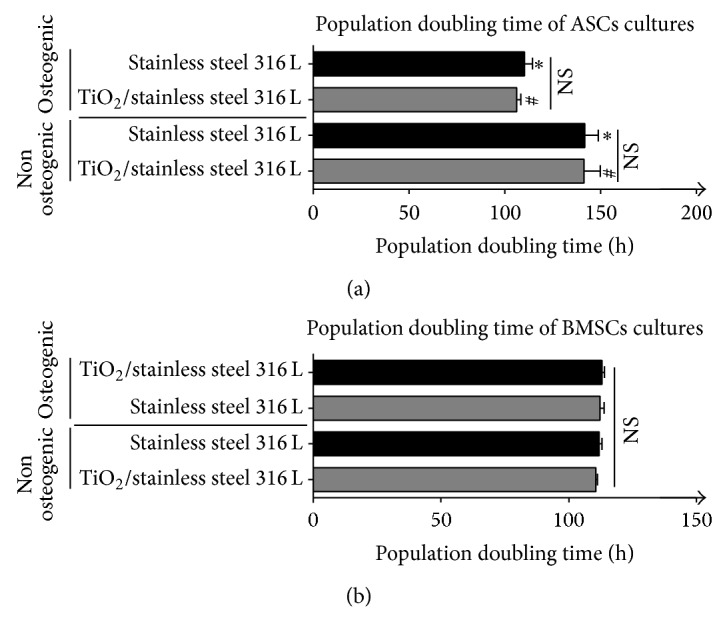
Comparison of population doubling time in osteogenic and nonosteogenic cultures of ASCs (a) and BMSCs (b) propagated on investigated biomaterials. Time needed for population doubling (PDT) was expressed in hours (h). Bars included in each column represent standard deviations (SD) calculated based on three measurements. Statistically significant differences were noted at *P* < 0.05. Significant differences were indicated using two marks: hashtag (#) for cultures on TiO_2_/stainless steel 316 L and asterisk (^*^) for cultures on stainless steel. Nonsignificant differences were indicated as NS. Analysis of differences between groups was tested using Student's *t*-test and two-way ANOVA.

The viability of cells was assessed in culture on the surface of both investigated biomaterials under osteogenic as well as nonosteogenic conditions. Determination of the population doubling time revealed that osteogenic culture medium accelerated the proliferation activity of ASCs, whereas activity of BMSCs was stable regardless of the cell culture conditions. However, the proliferation activity of BMSCs in the nonosteogenic culture was higher than ASCs. Analysis of population doubling time showed that proliferation of ASCs and BMSCs was not affected by the type of surface (Figure 3).

### 3.4. ALP Activity

**Figure 4:**
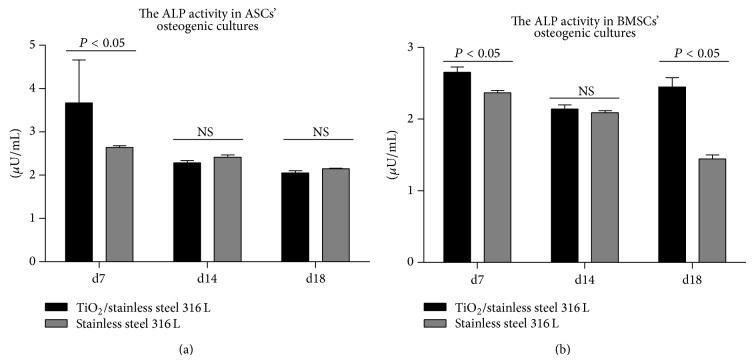
Effect of culture conditions on ALP activity in ASCs (a) and BMSCs (b) at days 7, 14, and 18. ALP activity shown in *μ*U/mL. Means and error bars (standard deviation values) are based on three measurements, each performed in duplicate. Indicated differences were significant at *P* < 0.05; lack of significance was marked as NS (not significant). Analysis was performed using two-way ANOVA test.

The examination of ALP activity was performed after 7, 14, and 18 days in osteogenic cultures. The significant increase of ALP activity in ASCs cultures was noted after 7 days of culture on biomaterial with TiO_2_ coating. Enzymatic activity of ALP measured after 14 and 18 days of ASCs cultures was comparable, regardless of culture conditions and surface used. Elevated activity of ALP in BMSCs cultures was noted when cells were propagated on stainless steel covered with TiO_2_ layer; however statistically significant dependencies were noticed after 7 days and 18 days of culture (Figure 4).

### 3.5. Quantitative Evaluation of Cellular Matrix Mineralization

**Figure 5:**
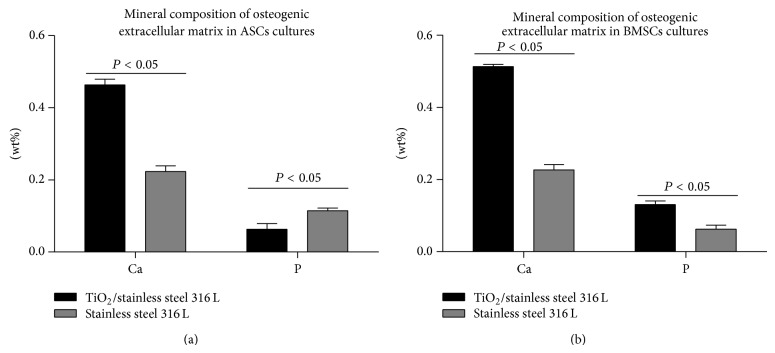
The result of elemental composition of extracellular matrix formed in osteogenic conditions in ASCs (a) and BMSCs (b) cultures. Calcium (Ca) and phosphorus (P) content were determined using SEM-EDX and shown as weight percentage (wt%). Means and error bars (standard deviation values) were calculated based on three measurements, each performed in triplicate. Indicated differences were significant at *P* < 0.05. Analysis was performed using two-way ANOVA test.

The quantitative measurement of calcium and phosphorus deposits in extracellular matrix (ECM) formed during osteogenic differentiation of ASCs and BMSCs was performed after 18 days of cells' propagation (Figure 5). Matrix rich in calcium deposits was produced both by ASCs and BMSCs cultured on stainless steel with TiO_2_ layer. In comparison to the cultures on pure stainless steel the amount of calcium in ECM of cultures on TiO_2_ sol-gel-derived biomaterial was significantly increased. The phosphorous content in ECM produced by BMSCs was also significantly elevated in cultures on TiO_2_; however in ASCs cultures ECM rich in phosphorus was noted when cells were propagated on pure stainless steel.

### 3.6. The Intra- and Extracellular Identification of OPN and OCL

**Figure 6:**
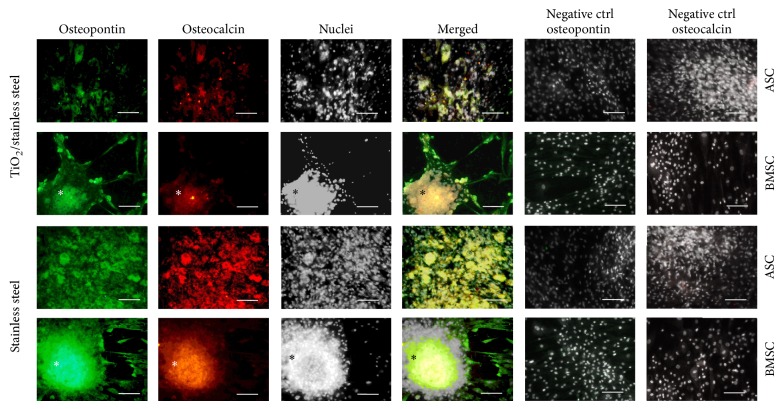
Micrographs presenting the immunofluorescence staining of BMSC and ASC after osteogenic differentiation on TiO_2_/stainless steel and pure stainless steel surface. Cell clusters marked with asterisks. Osteopontin shown in green, osteocalcin presented in red, nuclei shown in white; yellow areas on merged micrographs indicate the colocalization of both investigated proteins. Negative control staining was included in the picture. Scale bar = 100 *μ*m.

The intracellular expression of osteopontin and osteocalcin was detected in osteogenic cultures of ASCs and BMSCs, regardless of biomaterial used (Figure 6). The reactions noted were specific as was confirmed with negative control. The DAPI staining showed that the nuclei of cells were located centrally and had no signs of degeneration. Considerable differences between ASCs and BMSCs were related to the morphology and growth pattern. Biomaterials' surfaces were uniformly covered by the ASCs. The ASCs tightly adhered to each other forming active monolayer, whereas the cultures of BMSCs showed tendency for aggregation. The immunostaining reaction showed that within spheroid-like aggregates of BMSCs the expression of OPN and OCL was abundant (Figure 6).

**Figure 7:**
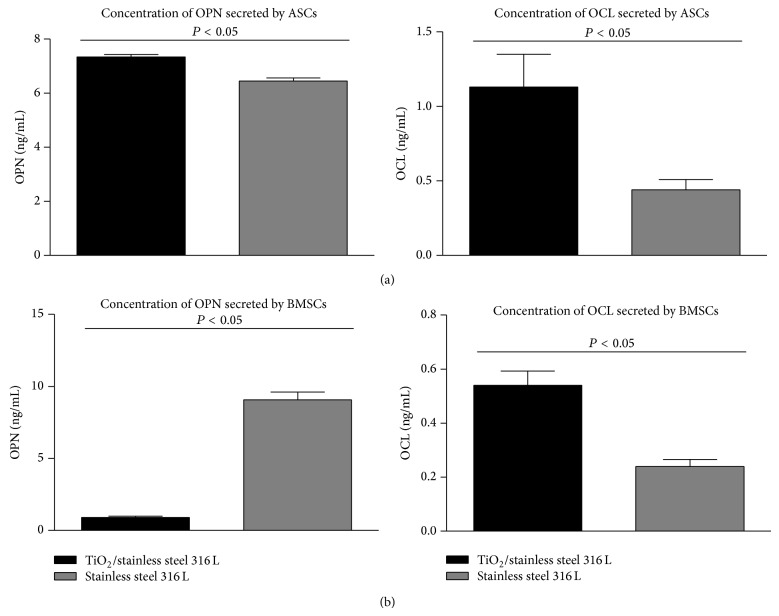
The concentration of OPN and OCL in the supernatants on day 18 of ASCs (a) and BMSCs (b) cultures. Means and error bars (standard deviation values) are based on three measurements, each performed in duplicate. Indicated differences were significant at *P* < 0.05. Analysis of differences was performed using Student's *t*-test.

Quantitative analysis of the OPN concentration showed that in ASCs cultured on biomaterial with TiO_2_ layer its level was significantly increased, whereas in BMSCs cultures reverse dependency was noted. Both investigated MSCs populations secreted increased amount of OCL, when cultured on stainless steel with TiO_2_ (Figure 7).

## 4. Discussion

Application of the sol-gel method to improve the biocompatibility of metallic implants continues to open new possibilities in the design of biomaterials. This technique brings special advantages in the development of biocompatibility properties of austenitic stainless steel. Diverse bioactive coatings are tested in order to enhance the corrosion resistance of metallic substrate and to ensure the optimal integration of the biomaterial with the surrounding tissue. Titanium dioxide layers synthesized with the sol-gel technique are often considered a biofunctional layer and a protective barrier against corrosion [23]. Titanium coatings obtained in this study had a homogenous topography. The microanalysis of their structure revealed that layers of TiO_2_ formed amorphous, transparent, and continuous coating. Such TiO_2_ films may give protection against corrosion and, in consequence, might extend the life of the prosthesis, which is a highly desirable feature in the material design [24]. Lim et al. showed that the titanium dioxide coating, deposited on stainless steel, promotes proliferation, alkaline phosphatase activity, migration, and adhesion of osteoblasts-like cells [25]. In our previous study, we showed that TiO_2_ coating applied onto stainless steel implant improves its integration with the bone tissue and limits the negative effects derived from foreign body response [12]. However, in this study, we decided to focus on a different angle of TiO_2_ biomaterial biocompatibility and to investigate the influence of titanium dioxide on the osteogenic differentiation of noncommitted multipotent mesenchymal stromal cells derived from adipose tissue and bone marrow. These two cell populations are readily used in the field of regenerative medicine; thus the choice of these cells for the study was natural [15, 16]. The main parameter used to describe the potential of multipotent mesenchymal stromal cells is their proliferation rate and cells' viability [26, 27]. The analysis of the proliferative activity of the cells showed that the biomaterials investigated did not exert cytotoxic effect on the MSCs selected for our experimental model. This result is consistent with many* in vitro* studies indicating that both 316 L and titanium dioxide substrates are biocompatible [25]. In our experiment, we have compared the proliferation activity of two model cell populations, that is, BMSCs and ASCs. In the context of proliferating activity, the population of BMSCs was more stable, both in the standard and in the osteogenic cultures. The proliferation activity of ASCs was accelerated when osteogenic factors were introduced to the culture.

In order to evaluate osteogenesis three markers were determined: ALP, OPN, and OCL. The ALP is considered as an early marker of osteogenesis, and actually its elevated production was noted both in ASCs and BMSCs after 7 days of culture on biomaterial with TiO_2_ layer. Enzymatic activity of ALP is also related to matrix mineralization during remodeling, which may explain the increased ALP production in BMSCs cultures after 18 days of culture [28]. The ALP activity is indicative of the matrix mineralization, but to evaluate the process of mineralization in a quantitative manner, after 18 days of culture, extracellular calcium and phosphorous deposits were detected using SEM-EDX. Obtained results showed that extracellular matrix produced by ASCs and BMSCs on the surface of TiO_2_ biomaterial was rich in calcium. Additionally in BMSCs cultures phosphorous concentration was elevated, which with increased activity of ALP brings evidence of favorable conditions for mineralization process. Results obtained are specific because the analysis of chemical composition of the TiO_2_ layer did not detect the presence of these elements; thus the Ca and P identified did not originate from the surface.

The osteogenesis process has also been proven by the expression of specific, noncollagenous proteins, osteopontin and osteocalcin, which are characteristic of the bone matrix formation. We decided to investigate intracellular as well as extracellular expression of these two osteogenic markers. The immunospecific staining showed that OPN and OCL are present intracellularly in ASCs and BMSCs, regardless of the properties of biomaterial surface; however complex relationships were noted when quantitative analysis of proteins secretion was performed. The production of osteopontin was increased in ASCs cultures significantly, when biomaterial with TiO_2_ layer was applied, while it decreased in BMSCs cultures. The osteopontin is a multifunctional protein and its expression may be connected with the proliferative activity of cells [29]. The ASCs in cultures on TiO_2_ surfaces in osteogenic condition indeed exhibited increased proliferation. Both ASCs and BMSCs revealed increased secretion of OCL in cultures on TiO_2_ layer, which additionally confirms osteopromoting properties of obtained biomaterial. Based on the quantity of the deposited Ca and P and high amount of released OPN, the osteogenesis alteration in ASCs might be induced spontaneously, as previously described in the literature [30]. The proliferation capacity of ASCs is higher than BMSCs [18]; however ability to formation of fully developed tissue is also crucial. The application of BMSCs for cell-based tissue engineering therapies is proposed as a suitable option due to the heterogeneity of this population reflecting on particular potential to differentiate into bone and bone-related cells [31]. An additional feature that may promote osteogenesis of BMSCs may be also their potential to form spheroid aggregates crucial at the initial stage of osteogenesis for inducement of functional bone formation [32]. As performed immunostaining showed, these aggregates are characterized by the high expression of OPN and OCL. Currently, ASCs are willingly used as an alternative source of multipotent stromal cells mainly due to easy access to the source tissue fat in contrast to bone marrow. Our results showed high proliferating activity of ASCs and formation of uniform active monolayer on biomaterial with TiO_2_ layer. These attributes combined with immunomodulatory potential of ASCS provide encouraging features in the context of tissue regeneration.

## 5. Conclusions

On the basis of our results indicating higher cellular stability of BMSCs, we conclude that this population may create more functional extracellular matrix when applied with TiO_2_ sol-gel-derived biomaterials and should be submitted for further* in vivo* studies. The TiO_2_ coating accelerated biological response of BMSCs to the osteogenic factors; however it did not influence their proliferation capacity. Strategy of combining BMSCs with TiO_2_ sol-gel biomaterials could bring new solution in bone tissue engineering and may provide significant advantages in improving the repair or replacement of damaged or even lost bone tissue. Nevertheless, we postulate to use both ASCs and BMSCs for* in vitro* assessment of biomaterials' cytocompatibility. Such approach may help to obtain comprehensive information about biological response of both cell populations and may extend area of investigations related to tissue engineering and regeneration therapy.
